# Atypical white dot syndrome with choriocapillaris ischemia in a patient with latent tuberculosis

**DOI:** 10.1186/s12348-018-0162-7

**Published:** 2018-11-03

**Authors:** Sana Khochtali, Nesrine Abroug, Imen Ksiaa, Sourour Zina, Sonia Attia, Moncef Khairallah

**Affiliations:** Department of Ophthalmology, Fattouma Bourguiba University Hospital, Faculty of Medicine, University of Monastir, Monastir, Tunisia

**Keywords:** Fluorescein angiography, Indocyanine green angiography, Optical coherence tomography angiography, White dot syndrome

## Abstract

**Background:**

White dot syndromes (WDS) are a group of idiopathic multifocal inflammatory conditions that can be recognized and distinguished by lesion morphology, other specific clinical features, imaging findings, and disease course. Our purpose is to describe an atypical case of WDS with choriocapillaris ischemia shown by multimodal imaging including swept-source OCT angiography (OCTA) in a 30-year-old woman with latent tuberculosis.

**Findings:**

At presentation, visual acuity in the left eye was 20/500. Clinical findings included macular granularity, diffuse outer retinal discoloration with satellite yellow-white dots, and disc margin blurring. Fluorescein angiography showed early confluent areas of choroidal hypofluorescence and late perifoveal punctate hyperfluorescence. There was choroidal hypofluorescence in a geographic configuration throughout the indocyanine green angiography sequence. OCTA showed confluent geographic areas of loss of signal in the choriocapillaris. Work-up revealed latent tuberculosis. The patient received corticosteroids and prophylactic anti-tubercular treatment. Nine months later, visual acuity had improved to 20/20, and there were some residual retinal pigment epithelium changes.

**Conclusion:**

Atypical WDS associated with choriocapillaris hypoperfusion may show features of multiple evanescent white dot syndrome and acute posterior multifocal placoid pigment epitheliopathy melting together.

## Introduction

The white dot syndromes (WDS) are a group of idiopathic multifocal inflammatory conditions involving the outer retina, retinal pigment epithelium (RPE), and/or choroid, usually occurring in young adults. They include multiple evanescent white dot syndrome (MEWDS), acute posterior multifocal placoid pigment epitheliopathy (APMPPE), serpiginous choroiditis, multifocal choroiditis/punctate inner choroidopathy/presumed ocular histoplasmosis syndrome, and others. These entities can be recognized and distinguished by lesion morphology, other specific clinical features, imaging findings, and disease course. There are however reports of WDS with atypical manifestations or overlapping clinical features [1].

We herein describe an atypical case of WDS with evidence of choriocapillaris ischemia on dye-based angiography and swept-source OCT angiography (OCTA) in a patient with latent tuberculosis.

## Case report

A 30-year-old woman was referred to our department with a 2-week history of sudden vision loss in the left eye. Her medical history was notable for fever and general malaise 2 weeks earlier. On examination, her best-corrected visual acuity was 20/20 in the right eye and 20/500 in the left eye. Pupils were equally round and reactive to light with no relative afferent pupillary defect. Results of anterior segment examination were unremarkable, and there were no vitreous cells in either eye.

**Fig. 1:**
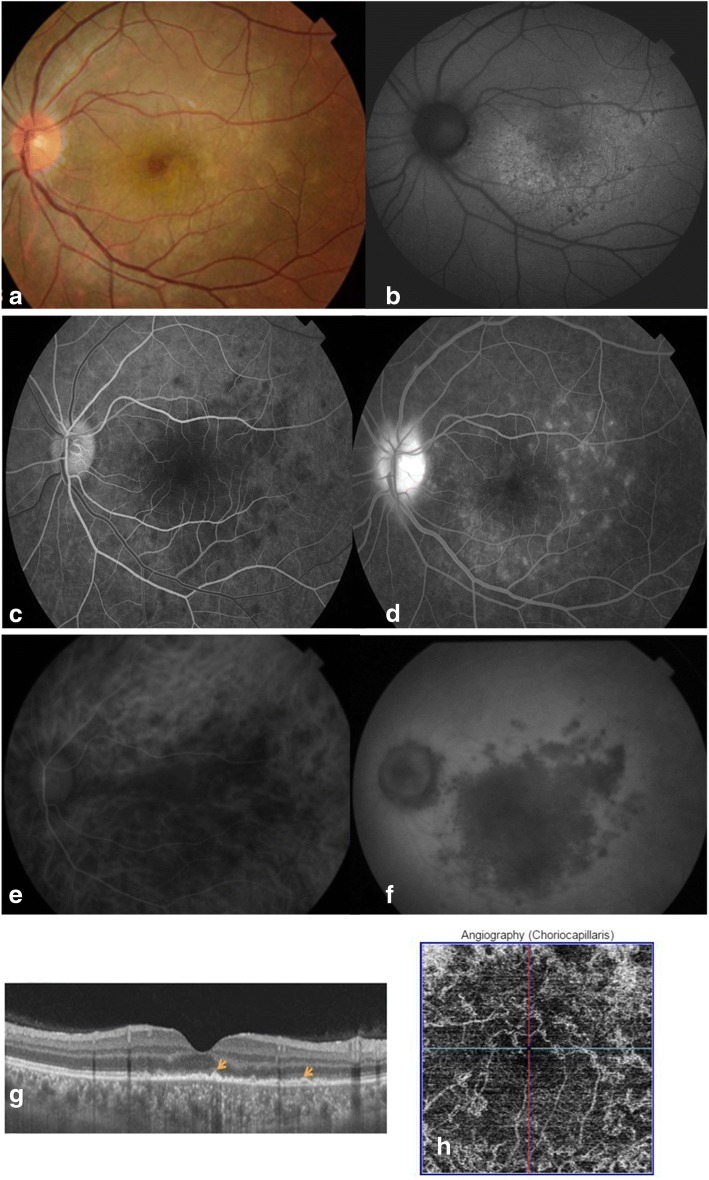
Multimodal imaging findings at presentation. **a** Fundus photograph of the left eye at presentation shows foveal granularity surrounded by diffuse retinal discoloration with satellite slightly indistinct, multifocal deep yellow-white dots. There also were blurred disc margins with peripapillary whitening. **b** Fundus autofluorescence of the left eye at presentation shows multiple, coalescent, punctate hyperautofluorescent lesions associated with focal small hypoautofluorescent areas. **c** Early-phase fluorescein angiogram of the left eye at presentation shows patchy areas of choroidal hypofluorescence in a geographic configuration. **d** Late-phase fluorescein angiogram demonstrates punctate hyperfluorescence with a “wreath-like” configuration around the fovea and optic disc leakage. **e** Early-phase indocyanine green angiogram of the left eye at presentation shows well-demarcated geographic areas of choroidal hypofluorescence that extend beyond the clinical limits of the yellowish retinal lesions, with large choroidal vessels visible within these hypofluorescent areas. This is highly suggestive of choriocapillaris ischemia and allows excluding a masking effect. **f** Late-phase indocyanine green angiogram shows persistent hypofluorescence in the posterior pole, with associated peripapillary hypofluorescence and optic disc staining. **g** Swept-source OCT image of the left eye at presentation shows disruption of the ellipsoid zone, accumulations of a hyperreflective material over the RPE (arrows), and hyperreflective dots in the inner choroid (subfoveal choroidal thickness = 370 μm). **h** Swept-source OCT angiogram of the choriocapillaris reveals confluent extensive geographic areas of loss of signal that colocalize with the ICGA hypofluorescent areas with associated projection artifact from retinal vessels

Dilated fundus examination of the left eye showed foveal granularity surrounded by diffuse deep yellow-white retinal discoloration with satellite slightly indistinct, multifocal deep yellow-white dots. There also were blurred disc margins with peripapillary whitening (Fig. 1a). The fundus of the right eye was unremarkable. Fundus autofluorescence (FAF) imaging of the left eye revealed multiple, coalescent, punctate hyperautofluorescent lesions associated with focal small hypoautofluorescent areas (Fig. 1b). Fluorescein angiography (FA) showed early confluent patchy areas of choroidal hypofluorescence and late punctate hyperfluorescence with a “wreath-like” configuration around the fovea and optic disc leakage (Fig. 1c, d). Indocyanine green angiography (ICGA) showed in the early and intermediate phase well-demarcated geographic areas of choroidal hypofluorescence in the posterior pole extending beyond the clinical limits of the yellowish retinal lesions that became more visible and more confluent in the late phase. Large choroidal vessels were visualized within these hypofluorescent areas, excluding any masking effect. There also were associated peripapillary hypofluorescence and optic disc staining indicating severe inflammation (Fig. 1e, f). Swept-source OCT (SS OCT) showed outer retinal layer changes including disruption of the ellipsoid zone, irregularities of the RPE, and accumulations of hyperreflective material resting on the RPE and extending anteriorly through the interdigitation zone, ellipsoid zone, and outer nuclear layer toward the inner retina. SS OCT also showed hyperreflective dots in the inner choroid and choroidal thickening (subfoveal choroidal thickness of 370 μm in the left eye vs 250 μm in the fellow unaffected eye) (Fig. 1g). Swept-source OCTA (DRI OCT Triton plus; Topcon) showed confluent extensive geographic areas of loss of signal in the choriocapillaris that colocalized with the ICGA hypofluorescent areas, highly suggesting a choriocapillaris ischemia (Fig. 1h). All imaging findings in the right eye were unremarkable.

Results of physical examination were normal. Laboratory tests, including a complete blood count, chemistry panel, serological test for syphilis, C-reactive protein, and erythrocyte sedimentation rate, were performed, all of which were within normal limits or negative. A chest X-ray was also unremarkable. Tuberculin skin test showed a 20-mm induration, and the result of QuantiFERON-TB Gold test was positive.

The patient received oral prednisone initiated with a dose of 1 mg/kg/day and then progressively tapered. The patient was also given isoniazid 300 mg/day and rifampin 600 mg/day for 3 months to treat latent tuberculosis.

**Fig. 2:**
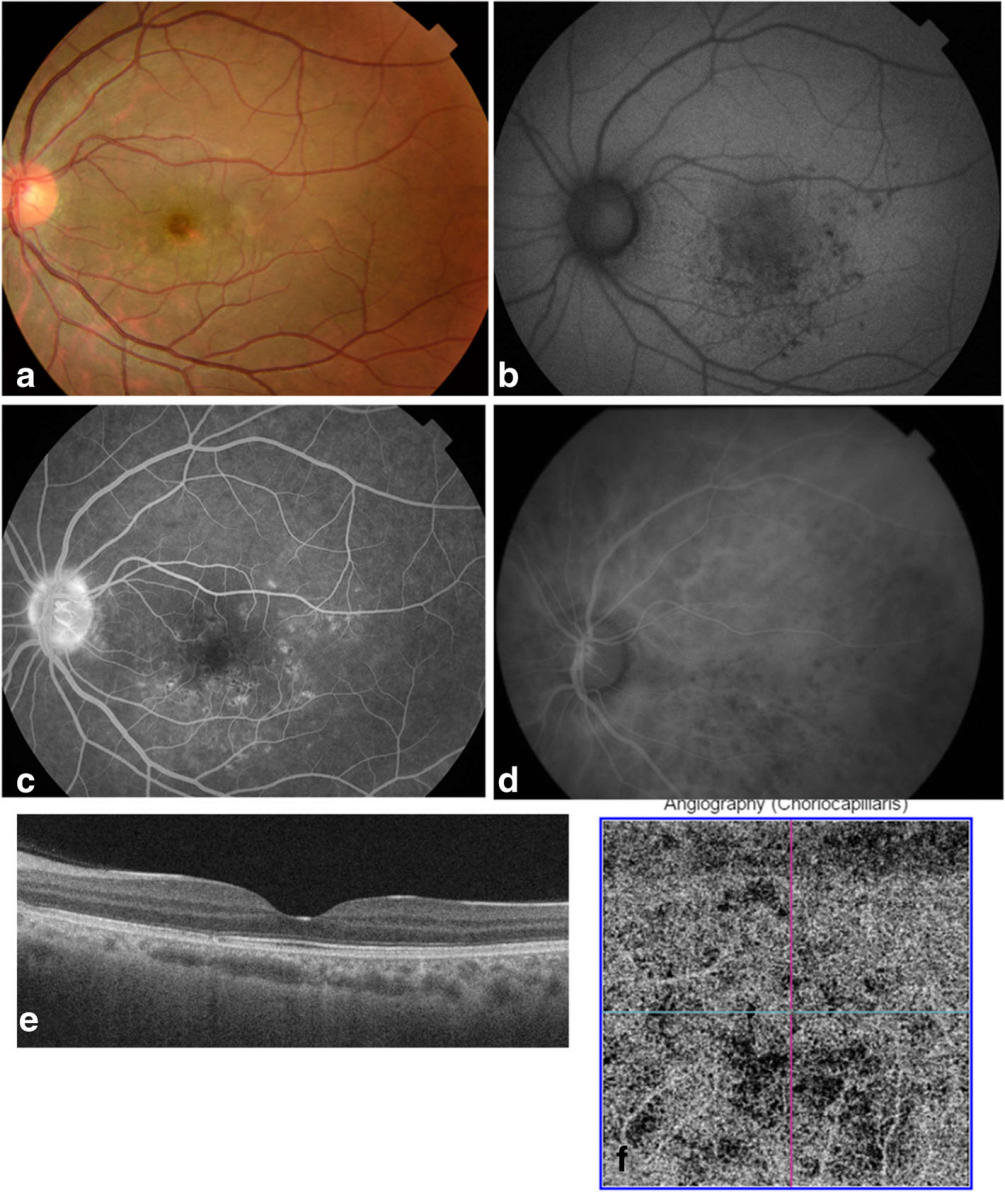
Multimodal imaging findings, 9 months after presentation. **a** Fundus photograph of the left eye, 9 months after presentation, shows some areas of RPE depigmentation. **b** Fundus autofluorescence image shows residual punctate areas of hypoautofluorescence. **c** Late-phase fluorescein angiogram demonstrates residual RPE changes. **d** Mid-phase indocyanine green angiogram shows small areas of hypofluorescence corresponding to residual scars on fluorescein angiography. **e** Swept-source OCT image of the left eye, 9 months after presentation, shows resolution of abnormal findings with recovery of a quite normal outer retinal and choroidal aspect (subfoveal choroidal thickness at 200 μm). **f** Swept-source OCT angiogram of the choriocapillaris reveals markedly improved areas of flow deficit

Nine months after initial presentation, visual acuity had improved to 20/20. The white retinal dots had completely resolved, leaving some areas of RPE depigmentation on FAF, FA, and ICGA (Fig. 2a–d). SS OCT showed complete resolution of abnormal findings with a recovery of a quite normal outer retinal and choroidal aspect (Fig. 2e). OCTA demonstrated markedly improved flow deficits of the choriocapillaris (Fig. 2f). The ocular findings remained unchanged over a further follow-up period of 12 months.

## Discussion

This young female presented with a complaint of unilateral acute central visual loss preceded by the onset of flu-like illness 2 weeks earlier. Clinical features exhibited by the patient including macular granularity, multiple deep white dots, and blurred optic disc margins in the absence of associated vitritis were consistent with a diagnosis of MEWDS [2, 3]. There also was a perifoveal diffuse outer retinal discoloration. Similar large circumpapillary, central, or peripheral geographic areas of retinal discoloration have been previously reported as a rare manifestation of MEWDS [2, 4, 5]. Such large geographic lesions probably represent a confluence of more typical MEWDS lesions, indicating a more severe disease, leading to severe loss of visual acuity in case of macular involvement, as seen in our patient [4, 6].

Our patient exhibited typical SS OCT changes of MEWDS including the disruption of the ellipsoid zone, and accumulation of hyperreflective material of variable size and shape, resting on the RPE and extending inward through the interdigitation zone, ellipsoid zone, and outer nuclear layer toward the inner retina, as well as choroidal hyperreflective dots and thickening [7, 8]. However, early extensive choroidal hypofluorescence in the macular area on FA and large macular hypofluorescence beginning from the early-phase of the ICGA highly suggesting choriocapillaris ischemia are unusual in patients with MEWDS [3, 9]. Extensive confluent macular areas of flow deficit in the choriocapillaris on swept-source OCTA in our patient are not consistent with data from a recent study that revealed a completely normal choriocapillaris flow on OCTA in all patients with MEWDS [10]. The rapid resolution of all acute clinical and imaging findings, with restoration of outer retinal layers on SS OCT and complete vision recovery are consistent with MEWDS in our patient. There however were residual small areas of RPE atrophy, a previously described finding in MEWDS, particularly in more severe disease [3, 7].

Other differentials that may be considered in our patient mainly include acute posterior multifocal placoid pigment epitheliopathy (APMPPE). Confluent deep plaques of yellow-white discoloration of the fundus may be seen in APMPPE. However, unlike the present case, lesions are typically more distinct, not associated with macular granularity and bilateral. The geographic hypofluorescent area present from the early to the late ICGA phase and early hypofluorescence on FA are also compatible with the diagnosis of APMPPE in our patient. In fact, initial hypofluorescence on ICGA and FA is due to the lack of choriocapillaris perfusion in typical cases of APMPPE. In our patient, the very extensive FA and ICGA hypofluorescence reflects the severity of choriocapillaris inflammation and ischemia. However, in APMPPE, there is typically a pronounced placoid hyperfluorescence in the late FA phase corresponding to the areas of hypofluorescence in the early FA, which was not observed in our patient [11]. Disruption of the outer retina and choroidal thickening on OCT, and areas of flow deficit on OCTA as evidenced in the present case have already been described as possible findings in APMPPE [12, 13]. But, the residual scars are typically more marked on FAF and FA in patients with APMPPE as compared to the reported case [11].

Of the battery of tests performed in our patient, tuberculin skin test showed a 20-mm induration and QuantiFERON-TB Gold test was positive. Active tuberculous posterior uveitis is a remote possibility in the present case. Posterior segment manifestations of tuberculosis mainly include vitritis, retinal vasculitis, optic neuritis, choroidal tubercles, serpiginous-like choroiditis, and choroidal tuberculoma. Serpiginous-like choroiditis also called multifocal serpiginoid choroiditis may simulate APMPPE. It usually presents as multifocal lesions showing a wave-like progression and/or a plaque-like choroiditis with amoeboid spread. It is usually associated with vitritis and is typically characterized by the coexistence in the same patient of active and healed chroidal lesions [14, 15]. None of the clinical features in our patient was consistent with serpiginous-like choroiditis. Ocular manisfestations were considered to be associated with but not related to latent tuberculosis, and the patient was given only prophylactic anti-tubercular treatment and not a full anti-tubercular regimen. The persistence of only mild residual retinal pigment epithelial changes and good visual outcome in this case further argue against serpiginous-like choroiditis [14, 15].

In summary, this case describes an atypical WDS associated with choriocapillaris hypoperfusion, confirmed by FA, ICGA, and swept-source OCTA. Signs of MEWDS overlap with manifestations of APMPPE in association with latent tuberculosis in the same patient. This case shows an intermediary severity between a typical MEWDS at one end of the spectrum and a typical APMPPE at the other end. Both WDS may represent the spectrum of the same disease involving the choriocapillaris, with different degrees of severity.

## Abbreviations

APMPPE: Acute posterior multifocal placoid pigment epitheliopathy
FA: Fluorescein angiography
FAF: Fundus autofluorescence
ICGA: Indocyanine green angiography
MEWDS: Multiple evanescent white dot syndrome
OCT: Optical coherence tomography
OCTA: Optical coherence tomography angiography
RPE: Retinal pigment epithelium
SS OCT: Swept-source OCT
WDS: White dot syndrome
